# Dr. Ahira R. White

**Published:** 1908-02

**Authors:** 


Dr. Ahira R. White died at his hime in Indianapolis, December 24, 1907, aged 65 years. He graduated from the medical department of the University of Buffalo in 1866.

